# Pseudo Muscular Hypertrophic Paralysis

**Published:** 1895-12

**Authors:** G. Manning Ellis

**Affiliations:** Professor of Anatomy in Chattanooga Medical College


For the Texas Medical Journal.

### PSEUDO MUSCULAR HYPERTROPHIC PARALYSIS.

BY G. MANNING ELLIS, M.D., PROFESSOR OF ANATOMY IN CHATTANOOGA MEDICAL COLLEGE.

Read before the Tri-State Medical Association of Georgia, Alabama and Tennessee.

THIS IS a rare predominately infantile disease, with an increase in the size or volume of the muscles, and accompanying loss of function, followed by paresis and atrophy.

Sir Chas. Bell, in 1830, first described, but did not recognize in this a distinct disease or separate it from muscular atrophy. Later it was accidentally discovered and recognized by Meryon, Dnchenne and Grisinger. It was Duchenne who published an account of it and gave it the name of pseudo-hypertrophic-muscular paralysis.

ETIOLOGY.—Males furnish the majority of cases, the proportion being about 4 to 1. The disease usually occurs in family groups and manifests itself early, usually at the close of infancy, but any time during development; rarely when growth is nearly ended. The first thing noticeable is the difficulty observed when the child first begins to walk. Children with this affection are usually later in attempting to walk, sometimes it being deferred until the fourth year. The impairment in power of locomotion is due to some degree of paresis, but the parents seldom notice this defect, because the child's muscles are large and seem well developed. Neither an increase or diminution of the muscles may be noticed for some time. The first muscles usually

*Bland Sutton. Diseases of Ovaries and Fallopian Tubes, p. 320.

affected are the sural or gastrocnemii. The child falls frequently, and there is inability to lift the heel, which makes it impossible for it to go up stairs. To differentiate this disease from rachitis request the child, while standing, to rise on the tip of its toes. The peculiarity of locomotion is an oscillating movement of the body from side to side, as each step is taken. To support itself, the child places the feet far apart; when walking, there is extreme lordosis. In the act of rising from a recumbent or sitting posture, if a support is near, the child climbs up on that; if not, he first gets up on his hands and knees, then grasps each thigh alternately and is at last enabled to get in an upright position. Beside the muscles of the calf, nearly all the muscles of the body may be affected—the extensors of the knee, the gluteal and lumbar muscles, latissimus dorsi, pectoral and biceps. Among the muscles of the trunk that stand out most conspicuously is the infra-spinatus. Following this increase in the muscles, we have them gradually diminishing or wasting; no trace of some muscles can be found after death. Among these the *teres major*, *pectoral and latissimus dorsi*. As a result of loss of the latissimus, the posterior axillary fold is absent, and shows a striking abnormality, especially when this contrasts with the enlarged infra-spinatus. The muscles supplied by the cranial nerves nearly always escape. Another important fact, there is no increase in strength as the muscles enlarge, but a degree of paresis precedes the hypertrophy, and the weakness continues to increase with the progress of the disease. The hypertrophy is always followed by atrophy. From this we have deformities of the spine; the *anterior posterior curve* with the concavity backward is one of the first symptoms of the disease. Besides the deformities of the spine, we have shortening and contraction of the flexors of the knee, elbow and ankle. The contraction in the knee and elbow occur late in the disease; the deformity of the ankle occurs earlier, because the muscles of the calf are first affected. The contraction coincides with the diminution in the bulk of the muscle. Electrical irritability is not much altered in the beginning of the disease, but is much lowered when the atrophic processes begin. The knee jerk at first is feeble and after a time disappears. The functions of the sympathetic system remain undisturbed. This disease rarely attacks girls; its great preponderance in males is one of its peculiarities. Hereditary transmission is always through the mother, through the ovum, a condition unknown in diseases of the nervous system. (*Gavero.*) The progress of the disease is variable. Between the 10th and 14th year, the power to stand is lost in a few cases, some being unable to assume an upright posture until after puberty. From the beginning of the hypertrophy in the calves, about eighteen months is required before the maximum of hypertrophy is attained; the disease then remains stationary for about two or three years, then an extension of the paralysis with hypertrophy goes to the trunk and upper extremities. (*Jacobi.*) After a complete loss of muscular power, the patient assumes a recumbent posture and finally dies with some pulmonary affection produced by an atrophy of the respiratory muscles.

PATHOLOGICAL ANATOMY.—The changes that take place in the affected muscles are most interesting. We have the muscular fibre changed from their primitive bundles, an hyperplasia gradually extending through them, followed by wasting, until the whole mass is chiefly composed of an overgrowth of connective tissue. Another alteration is, instead of the muscles being replaced by connective tissue, we have a deposition of fat. It is chiefly fat that causes the enlargement. There seems to be a tendency to this formation in the early stages of the disease. The contractions which occur are due to the absorption of the fat and atrophy of the muscular fibres.

Instead of the above conditions being separate, we usually have them combined; connective tissue and fat, with a few sarcolemma sheaths scattered through them, and an occasional bundle of muscular fibres. The motor nerves that supply the muscles have never been found to be diseased. In numbers of cases that have been examined the spinal cord has been found to be free from any lesion or changes. The normal condition of the anterior gray matter and motor nerve cells prove conclusively that the disease is not due to a pre-existing lesion of the spinal cord. It is a primary disease of the muscles consisting of an overgrowth of connective tissue with or without fat, followed by wasting of the muscular fibres. The muscles have been examined by excision during life, and portions have been excised by the "harpoon," an instrument devised by "*Duchenne.*" The fibres examined were found to be diminished to one-fifteenth or one-sixteenth their normal size. By the side of these were empty sarcolemma sheaths and hypertrophied fibres.

DIAGNOSIS.—The diagnosis of this disease is not difficult. The progressive character of it, taken with the age of the subject, the gradual diminution of strength in the lower extremities, the peculiarity of gait, the apparently well developed muscles, the mode of rising from the floor by placing the hands upon the knees, are almost absolutely pathognomonic of this disease. Infantile progressive muscular atrophy is easily excluded by first attacking the muscles of the face, especially the orbicular oris; the process of the disease extending downwards, pseudo hypertrophy invariably begins in the lower extremities and extends upward. Congenital spastic paraplegia would more easily be mistaken for it, but the patient does not rise from the floor in the way they do in pseudo-hypertrophy. In spastic paraplegia, the patient drags the feet after them, with the toes or ball of the foot constantly on the floor, being unable to lift the foot completely when walking. A constant spasm, with stiffness of the legs, when rising in the morning. The most important distinguishing diagnostic feature is the preservation and excess of the knee-jerk in spastic paraplegia. To differentiate it from spinal muscular atrophia, there is no difficulty. In this affection we have no enlargement of the muscles; the spinal form involves the muscles of the hand, rarely affecting the legs more than the upper extremities.

The prognosis of this disease is grave, and as each year passes we find a constant progression in the disability, and we know the patient can not reach adult life. Under all forms of treatment, the patient seldom recovers; a few cases have been recorded where the cure was complete.

Duchenne claims to have cured two cases; he treated them with massage, hydro therapeutics and faradization. Arsenic and phosphorus have been used to retard the progress of the disease, but no permanent arrest has been made by their use. Muscular exercise seems to retard its progress. Gymnastic exercises carefully managed so as to call into action all the muscles that need help should be arranged for the patient. The case I present I have had under treatment about two years. The mother noticed a difficulty in the child's first attempt to walk, but did not think strangely of this. But as he continued to grow and to walk clumsily and fall without apparent cause and rising with difficulty she consulted me. At this time it was impossible for him to walk upstairs, and he would assist himself by taking hold of the banisters. The child seemed perfectly developed but his oscillating gait, his apparent weakness in the calf muscles, and the characteristic peculiarity on rising from the floor confirmed my diagnosis. I could trace no history of this disease in either parent. I have used galvanism, given syr. iod. ferri. with minute doses of hg. $cl^2$, also have had the mother carefully rub and work the muscles of the thigh, leg and gluteal region two or three times a day, with some apparent benefit.

